# Sweetening Agents and Sweeteners in Dietary Supplements for Children-Analysis of the Polish Market

**DOI:** 10.3390/nu12082387

**Published:** 2020-08-09

**Authors:** Agnieszka Piekara, Małgorzata Krzywonos, Anna Szymańska

**Affiliations:** Department of Bioprocess Engineering, Wroclaw University of Economics and Business, room 301H, Komandorska 118/120, 53-345 Wrocław, Poland; malgorzata.krzywonos@ue.wroc.pl (M.K.); anna.szymańska@ue.wroc.pl (A.S.)

**Keywords:** sweetening agents, sweeteners, dietary supplements, children health

## Abstract

Sweetening agents (SA) and sweeteners are major additives used in the production of dietary supplements (DS), they fulfill both technological and organoleptic functions. The aim of this study is to identify the types of SA and sweeteners found in DS intended for children and to determine the secondary role of them. The study was performed on data from the documentation of representative samples of DS (N = 315) available on the Polish market. The results show that 75.24% of the products contained at least one SA or sweetener. Sucrose is the SA most frequently used in DS production. The empirical findings show that the type of sweetening ingredient correlates closely with the formulation of products, which in turn has to be suited to consumption abilities of the target group as well as to the children’s taste requirements. The crucial need for analysis of the composition of DS is emphasized in the light of high consumption rates of these products as well as limited regulations and policy.

## 1. Introduction

Sweetening agents (SA, e.g., sucrose, fructose, honey, molases) and sweeteners (e.g., cyclamate, aspartame, isomalt, mannitol, sorbitol, xylitol, erythritol) are among the most common food ingredients and additives. These ingredients are classified as “caloric” (e.g., sucrose, sugar alcohols and hydrogenated starch hydrolysates) or “non-caloric” (e.g., cyclamate, saccharin, aspartame, sucralose, acesulfame potassium, steviol glycoside) or respectively as “nutritive” or “non-nutritive” depending on their energy contribution [1]. Additionally, the term “natural origin” is used in the consumer information, as in the case of steviol glycoside, or “artificial” origin when they are synthetic [1]. Sweetening agents and sweeteners are often used in the production of non-alcoholic beverages [2,3], confectionery products [4], pickles, sauces [5] as well as foodstuffs applied to energy-restricted diets [6], dietary foods for special medical purposes or dietary supplements (DS) [7]. They are one of the reasons why the consumption of added sugars has risen dramatically over the past few decades and has negatively contributed to human health [3,8,9]. Due to the increasing epidemic of obesity [10,11] and number of individuals with diabetes [12,13], it is important to educate consumers to make reasonable and healthy food choices. The basis for the human diet, including diet of children, should consist of products in proportions consistent with the pyramid of healthy nutrition, possibly enriched foods and, ultimately, food supplements defined by Directive 2002/46/EC as foodstuffs the purpose of which is to supplement the normal diet and which are concentrated sources of nutrients or other substances with a nutritional or physiological effect [14]. The term “food supplement” was clarified in the above-mentioned Directive, whereas according to the Polish legislation products, those that are introduced into the market are labelled as dietary supplements. Consequently, the following paper uses this term. The directive has been the subject of considerable criticism because of its lack of sufficient regulation. Being a member of the European Union, Poland adopts EU laws. However, EU directives have only harmonized some issues, which relate, e.g., to the marketing of dietary supplements. Other key aspects remain within the competence of the member states. In Poland, the Chief Sanitary Inspectorate (Główny Inspektorat Sanitarny: GIS) is the governmental body responsible for granting an approval for sales of dietary supplements as foodstuffs. It is not difficult to introduce dietary supplements into the market, as the producer is only required to inform the GIS of the intention to market a product and to indicate the name of the product, its manufacturer, its nature, labeling, and its qualitative and quantitative composition. There are no Polish regulations preventing producers from introducing to market dietary supplements, which have been negatively evaluated, e.g., by the U.S. Food and Drug Administration. As a result, the rules governing the introduction of dietary supplements to the market cannot be perceived as strict [15]. Consumers who are more health conscious are more likely to use supplements because these products are associated with preventive health behaviour [16]. Media are also largely responsible for consumer perceptions of dietary supplements [17]. In recent years, there has been a growing number of studies on the use of dietary supplements by various user groups such as pregnant women [18,19], elderly people [20,21] or high school students [22] and artificial sweeteners knowledge among students [23]. Analyses of the consumption level of supplements indicate a high consumer interest in this range of products. While some studies have been conducted in this age group [24,25,26,27,28], more work is needed. The objective of our study is to indicate the types and define the role of sweeteners and sweetening agents found in dietary supplements for children between the ages of 3 and 12.

## 2. Materials and Methods 

The documentation consisting of product labels and information obtained from the manufacturers of 315 dietary supplements (herbal, minerals, multivitamins) available on the Polish market during the period from May 2016 to May 2017 formed the research material. Dietary supplements have been bought in pharmacies, drugstores and supermarkets in the city of Wroclaw (Poland) and in the on-line shops. The product specifications provided by the producers on the labels and leaflets including the list of ingredients, health and nutrition claims, were used. A database was created, which enabled effective comparison of selected supplements. The common denominators for selected group of products were: each product had declaration “dietary supplement” and was designed for children above 3 years of age; on the label could be found a clear statement that children are the target group of consumers, e.g., “for kids”. The recommended intake of dietary supplements varied from one to six portions. 

Among the 23 different identified forms of dietary supplements included in the studied product group, syrups, and syrups in capsules lozenges capsules they constituted together 48,89% A full list of forms includes: syrups, and syrups in capsules 23.17%, lozenges 13.97%, capsules 11.75%, jellies 9.52%, lollipops 8.57%, powders for dissolution in sachets or in straws 7.62%, liquids 7.30%, tablets for swallowing or chewing 6.69%, drops 2.22%, effervescent tablets 1.59%, chewable capsules 1.59%, dragee 1.27%, soluble gums 1.27%, pastilles 0.95%, candies 0.63%, chocolates 0.63%, spray 0.63%, jelly 0.32%, chewable tablets 0.32%. 

## 3. Results

Among 315 products studied, the largest group consisted of DS containing combinations of vitamins, minerals and plant compounds (herbal supplements), second constituted products containing mostly plant compounds (herbal supplements). The smallest group comprised products containing omega-3 fatty acids as well as probiotics.

Generally, according the division suggested by Gil–Campos [1] two groups, namely natural sugars and artificial sweeteners were identified after DS data had been studied (Table 1). Among each group, caloric and non-caloric agents were extracted. All polyols and high-intensity sweeteners are included in the list of substances permitted for use by Regulation (EC) No. 1333/2008 [29] (Table 1). However, there is a possibility that manufacturer’s material lists were not a true representation of the product’s content.

**Figure 1:**
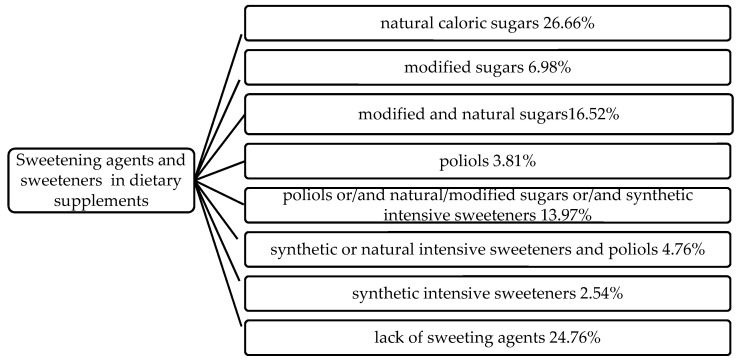
Combinations of sweetening agents and sweeteners in analyzed group of dietary supplements.

Among the studied products, 75.24% contained one or more sweetening agents and/or sweeteners. The other 24.76% of the supplements were mostly train oils and probiotics. Sucrose is the sweetener most frequently used in DS production and was among the ingredients of 41.91% of all products, where it was the only sweetening agent (18.10% of the products) or was used in combination with glucose syrup, high-fructose corn syrup, high-intensity sweeteners or polyols (Figure 1). 

EC Regulation No. 1169/2011 [30] stipulates that foods, including DS, must be labelled. The list of ingredients shall include all the components of the food in descending order of weight, as recorded at the time of their use in the manufacture of the food. By studying the product labels, it was found that a SA or sweeteners was the main ingredient of 53.97% of dietary supplements. Simple syrup (Sirupus simplex) consisting of 64.0 parts by weight of sucrose and 36.0 parts by weight of water is an input preparation (or an extract carrier) for flavouring, and/or improving the taste of medicine and dietary supplements in liquid form [31]. Among the products studied, 23.17% took the form of syrup, 83.56% contained sucrose as one of their ingredients. In DS, sucrose was replaced or supplemented with high fructose corn syrup, glucose syrup, polyols, fruit juices or honey, and thereby the supplement in syrup form did not have to be prepared on the base of simple syrup. In the DS group studied, only one type of syrup was prepared on a sugar base, which was emphasised on the label. None of the syrup types studied contained synthetic sweeteners among its ingredients.

In the studied DS group, 17.78% of the products contain one or more polyols while 4.76% of products contained steviol glycosides used in combinations with synthetic intensive sweeteners or polyols. Product which used only one artificial non-caloric sweetener (aspartame) consisted 2.54% of the entire studied group. A combination of sucralose and izomalt and/or xylitol appeared in 45.85 of the DS where polyols were used as a sweetener.

## 4. Discussion

Steviol glycosides extracted from Stevia rebaudiana have been used in Europe as sweeteners since November 2011 when Commission Regulation (EU) No. 1131/2011 permitted them for use in the European Union [32,33,34], which had been preceded by positive scientific opinion on their safety issued by the European Food Safety Authority [35]. In the studied DS group, 4.76% of products contained steviol glycosides used in combination with synthetic agents or polyols. Stevia (high-purity stevia leaf extract) is presented on the market as mixture with maltodextrins, erythritol or inulin and is considered to be a good sweetener in the prevention of obesity. Steviol glycosides are used commonly to reduce energy and added sugar content in food products [36]. Major food companies observed that consumers would pay more money for “natural” products. Thus, they made the decision to move away from artificial ingredients, which benefited the public health. Little research explores what messages are being communicated in DS advertising, and what persuasive tactics advertisers are using to influence consumers [17].

Due to the fact that up to 50% of children are likely to take dietary supplements, caretakers should be aware of the kinds and amount of SA presented in a DS (especially when more than 40% of these products include sucrose). Consumers show great confidence/trust in the safety of dietary supplements and may therefore not see the possible side effects of their use, especially among children [37]. Due to the lack of regulations imposing the obligation to inform consumers about the caloric value of supplements as well as the content of sugars, parents may not realize that the daily dose of supplement (containing one to six portions) may be a source of significant amounts of sugar in their children’s diet, e.g., six lollipops of 10g per piece in whose main ingredient is sucrose [38,39]. In addition, it is worth mentioning that the more fillers that were used to produce a supplement (mainly in chewing gums, jellies, lollypops, candy), the less “space” there was for active ingredients [40]. 

However, sweetening agents such as sucrose, glucose, and fructose have an influence on oral hygiene. When used in high amounts, the risk of dental caries (tooth decay) in children increased [41,42]. The opposite effect is caused by both Xylitol and less so by other polyols [43,44]. However, it is only so if they are not used in the supplements along with sweetening agents. Moreover, animal studies confirmed that the consumption of sweeteners might result in metabolic dysregulation [45,46], whereas in humans, they have been associated with weight gain and diabetes [47,48].

The analysis of the composition of the studied dietary supplements showed that 17.78% of the products contain one or more polyols. Sugar alcohols are widely used in food, beverage, confectionery and pharmaceutical industries throughout the world. They are added to foods as alternative sweeteners that might be helpful in the control of calorie intake [9]. Polyols enable the confectioner to develop suitable sugar-reduced and non-sugar alternatives. These products are chemically defined as saccharide derivatives in which a ketone or aldehyde group is replaced by a hydroxyl group and include sorbitol, mannitol, maltitol, lactitol, isomalt, xylitol and erythritol [49]. 

Polyols play a big role in DS manufacturing technology. Owing to their lower sweetening intensity than that of sucrose, these sweeteners serve as fillers (to increase the volume of a product and at the same time to reduce the unit calorific value of the product) [1,50]. For food labelling purposes, the European Union has agreed that in calculating the energy value of food, the calorific value of all polyols shall be 2.4 kcal/g compared to a value of 4 kcal/g for sugars and other carbohydrates, so polyols can be used to replace some or all of the sugars [49]. They are chemically stable, and therefore they are used as carriers. In addition, they bond certain macroelements, and for this reason they are added as stabilisers to mineral and vitamin preparations present on the market in the form of jellies or lozenges [51]. An addition of sorbitol increases the shelf life of products such as chewing gums, pills and lozenges, by preventing the contained sucrose from re-crystallising. Apart from the sweetening function, it was noted that polyols also performed other functions in 11.43% of the analysed group of products. The technological function of each of the polyols contained in dietary supplements can be described as follows:

- Sorbitol—a humectant: chewing gums, jellies, lozenges;
- Sorbitol—a filler/carrier: jellies, effervescent tablets, lozenges, sachets;
- Sorbitol—a stabiliser: jellies, lozenges;
- Xylitol—a filling compound: lozenges;
- Isomalt—a filling compound: lozenges, lollipops;
- Maltitol—a filling compound: liquid.

An expected sweet taste of a product can be generated by adding a several-times-smaller amount of high-intensity sweeteners than it would have been the case if sucrose was used. This is caused by much higher sweetening strengths of sweeteners compared to sucrose. Using suitable agents, one can reduce the unpleasant taste of bitter actives. A universally acceptable taste-masking technology does not seem to exist. Yet, the aversion to bitter taste is universal. Many current taste masking efforts are directed at reducing the negative attributes of paediatric dosage forms, which is a big challenge [52]. Moreover, sweeteners have technologically useful properties such as desired shelf life, texture or the property of masking undesirable tastes and of strengthening flavours [53]. The studied group of dietary supplements for children included the products where high-intensity sweeteners were added in mixture form, and synergy effects, or enhanced sweetening strengths, were observed. A synergy effect occurred when a mixture of polyols and a mixture of polyols and high-intensity sweeteners were applied [50]. A mixture of sucralose and isomalt and/or xylitol occurred most frequently; it was identified in 45.83% of the products where polyols were used as sweetener.

Aspartame is universally considered as a fruit flavour enhancer [54], but it was used as a sweetener in 2.54% products out of those studied, including only one fruit-flavoured dietary supplement. Acesulfame K (ace-K) is equally rarely met in dietary supplements for children—only 3.15% of products contained ace-K (each time in combination with sucralose or aspartame). Ace-K demonstrates its sweet flavour in a narrow range of concentrations only; if the concentration limits are exceeded, a bitter or metallic aftertaste can be felt, which limits its use [55]. A decidedly wider application to DS manufacturing technology was shown by sucralose, which occurred among the ingredients of 10.48% products (where a synergy effect caused by combinations of appropriate polyols and high-intensity sweeteners was also used) [50]. While aspartame is metabolised, insulin levels in the blood do not increase. For this reason, products containing aspartame can be consumed by individuals with diabetes [56]. In the case of sucralose, the available evidence indicates that this sweetener is safe for its intended use including for both normoglycemic and diabetic individuals [57,58], however, a study of Pepino et al. on a small and non-blinded population demonstrated possible enhancing insulin secretion by sucralose [56]. High-intensity sweeteners, both synthetic (aspartame, ace-K, sucralose, cyclamates) and natural (steviol glycosides) are universally used in the production of DS, however, no synthetic sweeteners should be used to make products for children under 3 years of age [33]. Owing to their much higher sweetening strengths than that of sucrose, sweeteners can be added in much smaller quantities. 

The technological and economic (application of high-intensity sweeteners results in lowering the costs) reasons are not the only ones that have an influence on the diversity of SA and sweeteners that are used in DS. Medical contraindications in the consumption of certain additions are also an important reason for their use. This applies to individuals with diabetes, persons with diagnosed phenylketonuria and food intolerance (e.g., fructose intolerance). Table 2 contains a summary of the contraindications that should prevent children from consuming products with certain sweeteners used in DS. It is important to indicate that in many cases (health conditions) selected dietary supplements should be avoided. It also points out that parents should prevent children from consuming products with certain ingredients

All studies have limitations. The major limitations of this study that could be addressed in future research are, firstly, time limits—the analysis of the products was carried out in a specific time. However, the authors are continuing their research on this segment of the DS and no immediate changes in the market structure of these products have been observed. Secondly, the authors understand that producers change their products, improve compositions and modify the ingredient lists.

## 5. Conclusions

Caloric sweetening agents and sweeteners play an important role in the production of DS, the use of which, without a doubt, correlates with the form of the product (e.g., lozenges, jellies, lollipops). The technological and organoleptic reasons for using these substances are obvious. However, the ingredients such as sucrose or glucose syrup can make up more than 90% of DS mass, as in the case of the lollypops [39]. This group of SA and the health consequences of their consumption such as obesity or dental caries has been well described in literature [60], whereas in the diet of children, dietary supplements, next to sweetened beverages and sweets, are one of the sources of SA [61]. Taking into account the negative effects of their consumption, one should consider introducing restrictions in the production of supplements for children with caloric sweetening agents. Consumption of low-calorie foods by the worldwide population has increased dramatically, and so have health concerns associated with the consequent high intake of SA [10]. Supplementation is perceived by consumers as an easy way of improving nutritional status without changing their diet, as such a change might require some effort. The provision of supplements to school-age children is a rather common phenomenon in many regions [62]. The range of DS available on the market is undoubtedly adjusted to meet children’s expectations. The preparations that are sweet in taste are readily taken by children, all the more when a product is offered in the form of conventional sweets. A significant market share (56%) of the products that are based on natural sugars (sucrose, sugar syrups) resulting from the need to use sugar for technological reasons, to obtain desired product forms such as syrups, lozenges, and lollipops. If children take supplements, the amount of sugars consumed needs to be under the control to prevent dental caries and obesity, as there is a noticeable tendency among children to consume too much sugar in their everyday diet [63]. It can also be found that the dietary supplement manufacturers are interested in using substitutes such as steviol glycosides and xylitol. This is a positive trend that leads to the reduction of the calorific value of a supplement. Moreover, the diversity of sweetening additions available on the market makes it possible to prepare supplements for various groups of consumers who suffer from intolerances (fructose intolerance) or diseases (individuals with diabetes). The real necessity of supplementation of the children’s diet should be evaluated. Specific population groups should be targeted by public health initiatives to prevent the possible side effects of the intake of DS, which definitely affect quality of life.

## Figures and Tables

**Table 1:** Sweetening agents and sweeteners in analyzed group of dietary supplements.

Natural Sugars			Artificial Sweeteners		
Caloric		Non-Caloric	Caloric		Non-Caloric
Sugars	Others	Natural intensive sweeteners	Modified sugars	Sugar alcohols	Synthetic intensive sweeteners
sucrose sugar cane glucose fructose	honey agave syrup tapioca tapioca syrup	Steviol glycosides	glucose syrup D-mannose isomaltulose (High fructose) corn syrup, inverted sugar	sorbitol, xylitol, mannitol maltitol izomalt sorbitol syrup	acesulfame K aspartame sodium saccharin sucralose sodium cyclamate

**Table 2:** Contraindications in the consumption of products with certain sweetening agents and sweeteners present in dietary supplements for children [1,59].

Sweetening Agents and Sweeteners	Contraindication
Sucrose, glucose, glucose–fructose syrup	Individuals with diabetes
Fructose, sorbitol, glucose–fructose syrup	fructose intolerance
Sorbitol and other polyols	Children under 6m because of the risk of diarrhea
Honey	Allergy to honey and pollen; diabetes
Synthetic intense sweeteners	It is inadvisable to use for children under 3 years of age
Aspartame	It is inadvisable to use for children under 3 years of age Phenylketonuria
